# Novel risk group stratification for metastatic urothelial cancer patients treated with immune checkpoint inhibitors

**DOI:** 10.1002/cam4.2932

**Published:** 2020-02-25

**Authors:** Julie M. Shabto, Dylan J. Martini, Yuan Liu, Deepak Ravindranathan, Jacqueline Brown, Emilie E. Hitron, Greta A. Russler, Sarah Caulfield, Haydn Kissick, Mehrdad Alemozaffar, Kenneth Ogan, Wayne B. Harris, Viraj A. Master, Omer Kucuk, Bradley C. Carthon, Mehmet A. Bilen

**Affiliations:** ^1^ Department of Hematology and Medical Oncology Emory University School of Medicine Atlanta GA USA; ^2^ Winship Cancer Institute of Emory University Atlanta GA USA; ^3^ Departments of Biostatistics and Bioinformatics Emory University Atlanta GA USA; ^4^ Department of Medicine Emory University Atlanta GA USA; ^5^ Department of Pharmaceutical Services Emory University School of Medicine Atlanta GA USA; ^6^ Department of Urology Emory University School of Medicine Atlanta GA USA

**Keywords:** cancer risk factors, immunology, risk assessment, urological oncology

## Abstract

**Background:**

We developed a novel risk scoring system for urothelial cancer (UC) patients receiving immune checkpoint inhibitors (ICI).

**Methods:**

We conducted a retrospective review of 67 UC patients treated with ICI at Winship Cancer Institute of Emory University from 2015 to 2018. Using stepwise variable selection in Cox proportional hazard model and Sullivan's weighting schema, baseline platelet‐to‐lymphocyte ratio (PLR), presence of liver metastasis, baseline albumin, and baseline Eastern Cooperative Oncology Group performance status (ECOG PS) were used for risk scoring. Patients were categorized into good risk (risk score 0‐1), intermediate risk (risk score 2‐3), and poor risk (risk score 4‐6). Univariable (UVA) and multivariable analysis (MVA) and Kaplan‐Meier method were used to assess overall survival (OS) and progression free survival (PFS).

**Results:**

The Emory Risk Scoring System had C‐statistics of 0.74 (Standard Error = 0.047) in predicting OS and 0.70 (Standard Error = 0.043) in predicting PFS. Compared to good risk patients, poor risk patients had significantly shorter OS and PFS in both UVA and MVA (all *P* < .001), and intermediate risk patients had significantly shorter OS and PFS in both UVA and MVA (all *P* < .03).

**Conclusions:**

Risk scoring using baseline PLR, presence of liver metastasis, baseline albumin, and baseline ECOG PS may effectively predict OS and PFS in UC patients receiving ICI.

## Graphical abstract

Novel Emory risk scoring system may predict clinical outcomes in urothelial cancer patients receiving immunotherapy. Increased baseline PLR, low baseline albumin, metastasis to the liver, and higher ECOG PS associated with decreased survival.

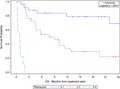

## 1 BACKGROUND

Immune checkpoint inhibitors (ICI) have emerged as promising treatment options for patients with various primary cancer histologies including melanoma, lung cancer, renal cell carcinoma, and urothelial cancer (UC). ICI agents have a tolerable toxicity profile and offer the promise of durable responses.^1^, ^2^, ^3^ Several ICI agents have been approved over the past 4 years by the FDA for treatment of patients with metastatic UC, including nivolumab, atezolizumab, pembrolizumab, avelumab, and durvalumab.^4^Unfortunately, a subset of patients still does not respond to ICI, and immune‐related adverse events, although rare, can significantly affect patients’ quality of life.^3^, ^5^ Hence, it is very important to find biomarkers of response for UC patients treated with ICI.

At this point, there is no universally accepted risk stratification system for UC patients receiving ICI. Sonpavde et al (2016) showed that albumin, hemoglobin, performance status, presence of liver metastasis, and time from previous chemotherapy were significant prognosticators of overall survival (OS) in UC patients treated with salvage systemic therapy.^6^ However, patients treated with ICI likely require unique risk stratification given that ICI rely on the reaction of the host immune system for their response.^7^

In this study, we investigated the factors most predictive of clinical outcomes in UC patients treated with ICI at our institution. We developed a novel risk stratification system, the Emory Risk Scoring System for UC patients treated with ICI, using four risk factors: platelet‐to‐lymphocyte ratio (PLR), Eastern Cooperative Oncology Group performance status (ECOG PS), presence of liver metastasis, and albumin. Using these aforementioned four variables as proxies for systemic inflammation, clinical presentation, tumor microenvironment, and nutritional status, respectively, we categorized the patients into three risk groups with regard to relevant clinical outcomes.

## 2 MATERIALS AND METHODS

### 2.1 Data collection

We conducted a retrospective review of 67 UC patients treated with PD‐1 or PD‐L1 inhibitors at Winship Cancer Institute of Emory University between 2015 and 2018. OS and progression free survival (PFS) were measured from start of ICI to date of death or hospice referral and clinical or radiographic progression, respectively.^8^ Several variables at baseline were collected from electronic medical records including demographic information, monocyte‐to‐lymphocyte ratio (MLR), neutrophil‐to‐lymphocyte ratio (NLR), PLR, albumin level, hemoglobin level, ECOG PS, number and sites of distant metastases, and body mass index (BMI). Sites of metastasis were collected from radiology reports and clinical notes. BMI was used to represent body composition.

### 2.2 Statistical methods

All data analyses were done in SAS 9.4 with summary reports generated by SAS macros.^9^ Summary statistics were applied to all variables of interest. Univariable analysis (UVA) of the association between collected variables and OS and PFS used Cox proportional hazard model. For continuous biomarkers, their nonlinear relationship with OS was examined by martingale residual plot and an optimal cutoff (OC) that maximizes the separation between the two groups was searched by a bias adjusted log rank test.^10^, ^11^

Using the significant prognostic factors per UVA, a stepwise variable selection was implemented in Cox proportional hazard model regarding OS with entering *P* < .3 and staying *P* < .1. Based on the final prediction model, a score was assigned according to the Sullivan's weighting schema, where the regression coefficient (RC) for each predictor was divided into the smallest absolute RC for all predictors and rounded to the nearest integer.^12^, ^13^

The Emory Risk Scoring System for UC patients treated with ICI is shown in Table 1. The final variables selected for the risk scoring system were baseline PLR, presence of liver metastasis, baseline albumin level, and ECOG PS. The optimal cut for PLR and albumin were 301.87 and 3.9 g/dL, respectively. We rounded the PLR optimal cut value of 301.87‐302 for ease in using the risk scoring system. The variables baseline PLR ≥302 and presence of liver metastasis each counted as 1 point in the risk score, while baseline albumin ≤3.9 g/dL and baseline ECOG PS ≥2 counted as 2 points each. Based on the 6‐month and 12‐month survival rates and sample size distribution for each individual score of 0 through 6, patients were further stratified into good risk (risk score 0‐1), intermediate risk (risk score 2‐3), and poor risk (risk score 4‐6). The Cox proportional hazard model was used for the related survival analysis for OS in the univariable and multivariable models. Kaplan‐Meier method was applied to determine the median OS and PFS for each risk group. The discrimination power by the Emory Risk Scoring System in predicting survival was measured by Uno's C‐statistics.^14^

**Table 1:** Emory risk scoring system for UC patients treated with immune checkpoint inhibitors

Variable	Points
PLR ≥302	1
PLR <302	0
Liver metastasis	1
No liver metastasis	0
Albumin <3.9 g/dL	2
Albumin ≥3.9 g/dL	0
ECOG PS ≥2	2
ECOG PS <2	0
*Total possible*	*6*

## 3 RESULTS

### 3.1 Patient characteristics

Descriptive statistics of this patient cohort are presented in Table 2. The median patient age was 69 and most (79%) were male. Many patients (42%) had received two or more prior lines of systemic therapy before receiving ICI. Sites of metastasis collected were lymph node (n = 49), lung (n = 21), bone (n = 20), liver (n = 14), and brain (n = 1). Most patients (88%) had ECOG PS of 0 or 1 at baseline. Patient BMI ranged from 15.8 to 49.5, with a mean of 26.3. Patients were on treatment for an average of 28.1 weeks, with a range of 2.9 to 131 weeks. Median baseline NLR was 4.00, median baseline MLR was 0.58, and median baseline PLR was 193.08.

**Table 2:** Baseline patient characteristics

Variable	N (%) = 67
Median age	69 (range: 32‐93)
Sex	
Male	53 (79)
Female	14 (21)
Race	
White/Asian	54 (81)
Black	13 (19)
Eastern cooperative oncology group performance status	
0	45 (67)
1	14 (21)
2‐3	8 (12)
Smoker	
Yes	33 (49)
No	34 (51)
Number of metastatic sites	
0‐1	27 (40)
2	24 (36)
3‐5	16 (24)
Site of metastasis	
Lymph node	49 (73)
Lung	21 (31)
Bone	20 (30)
Liver	14 (21)
Brain	1 (2)
Number of prior systemic therapies	
0‐1	39 (58)
2	14 (21)
3‐5	14 (21)
Type of immunotherapy	
Atezolizumab	50 (75)
Pembrolizumab	12 (18)
Nivolumab	3 (4)
Nivolumab + experimental agent	2 (3)

### 3.2 Emory risk group analysis

All collected variables were examined for their association with OS and PFS using Cox proportional hazard model. Results of the UVA of a sample of the variables we explored are shown in Table 3. The UVA and multivariable analysis (MVA) of the association between the Emory risk groups and survival is presented in Table 4. In UVA, poor risk patients had significantly shorter OS (HR: 169.39, CI: 34.94‐821.24, *P* < .001) and significantly shorter PFS (HR: 43.65, CI: 13.65‐139.60, *P* < .001) compared to good risk patients. In MVA, poor risk patients had significantly shorter OS (HR: 230.79, CI: 44.26‐1203.52, *P* < .001) and significantly shorter PFS (HR: 38.46, CI: 11.93‐123.99, *P* < .001) compared to good risk patients. Intermediate risk patients also had significantly shorter OS and PFS compared to good risk patients in both UVA and MVA (all *P* < .03).

**Table 3:** UVA of explored covariates with survival

Variable		OS		PFS	
		HR (CI)	*P*‐value	HR (CI)	*P*‐value
ECOG PS	0‐1 (n = 59)	0.28 (0.12‐0.69)	**.005** *	0.34 (0.15‐0.76)	**.009** *
	2‐3 (n = 8)	—	—	—	**—**
Number of metastatic sites	0‐1 (n = 27)	0.41 (0.16‐1.02)	.055	0.49 (0.23‐1.04)	.064
	2 (n = 24)	1.22 (0.55‐2.72)	.628	0.99 (0.50‐1.98)	.980
	3‐5 (n = 16)	—	—	—	**—**
Prior lines of therapy	0‐1 (n = 39)	1.34 (0.57‐3.13)	.504	0.94 (0.47‐1.89)	.871
	2 (n = 14)	0.99 (0.35‐2.83)	.983	0.67 (0.28‐1.62)	.373
	3‐6 (n = 14)	—	—	—	**—**
Sites of metastasis	No lymph mets (n = 18)	0.94 (0.45‐1.95)	.873	0.70 (0.36‐1.37)	.296
	Lymph mets (n = 49)	—	**—**	—	**—**
	No bone mets (n = 47)	0.38 (0.19‐0.73)	**.004** *	0.46 (0.25‐0.82)	**.009** *
	Bone mets (n = 20)	—	**—**	—	**—**
	No liver mets (n = 53)	0.41 (0.20‐0.85)	**.017** *	0.56 (0.29‐1.11)	.096
	Liver mets (n = 14)	—	**—**	—	**—**
	No brain mets (n = 66)	0.67 (0.09‐4.94)	.696	1.36 (0.19‐9.87)	.762
	Brain mets (n = 1)	—	**—**	—	**—**
	No lung mets (n = 46)	1.08 (0.53‐2.19)	.825	0.93 (0.51‐1.68)	.803
	Lung mets (n = 21)	—	**—**	—	**—**
Baseline albumin	≥3.9 g/dL (n = 46)	0.23 (0.11‐0.46)	**<.001** *	0.36 (0.20‐0.65)	**<.001**
	<3.9 g/dL (n = 21)	—	**—**	—	**—**
Baseline Hgb	≥10 g/dL (n = 53)	0.45 (0.22‐0.93)	**.030** *	0.54 (0.28‐1.02)	.057
	<10 g/dL (n = 14)	—	**—**	—	**—**
Baseline BMI	<25 (n = 27)	1.19 (0.62‐2.31)	.603	1.05 (0.59‐1.85)	.873
	≥25 (n = 40)	—	**—**	—	**—**
Sex	Female (n = 14)	0.91 (0.40‐2.07)	.816	0.93 (0.47‐1.87)	.849
	Male (n = 53)	—	**—**	—	**—**
Baseline PLR at optimal cut (301.87)	Below (n = 49)	0.30 (0.15‐0.58)	**<.001** *	0.45 (0.24‐0.81)	**.008** *
	Above (n = 18)	—	**—**	—	**—**
Baseline NLR at optimal cut (4.66)	Below (n = 38)	0.29 (0.15‐0.58)	**<.001** *	0.52 (0.30‐0.91)	**.023** *
	Above (n = 29)	—	**—**	—	**—**
Baseline MLR at optimal cut (0.55)	Below (n = 33)	0.40 (0.20‐0.79)	**.008** *	0.64 (0.37‐1.13)	.128
	Above (n = 34)	—	**—**	—	**—**

Bold values are statistically significant with *α* < 0.05. Abbreviations: BMI, body mass index; CI, confidence interval; ECOG PS, Eastern Cooperative Oncology Group Performance Status; Hgb, hemoglobin; HR, hazard ratio; Mets, metastasis; MLR, monocyte‐to‐lymphocyte ratio; NLR, neutrophil‐to‐lymphocyte ratio; OS, overall survival; PFS, progression free survival; PLR, platelet‐to‐lymphocyte ratio; UVA, univariable analysis.

* Statistical significance at α < 0.05.

**Table 4:** UVA and MVA^a^ of risk group and survival

Risk groups	UVA				MVA			
	OS		PFS		OS		PFS	
	HR (CI)	*P*‐value	HR (CI)	*P*‐value	HR (CI)	*P*‐value	HR (CI)	*P*‐value
Poor risk (score = 4‐6) n = 9	169.39 (34.94‐821.24)	**<.001** *	43.65 (13.65‐139.60)	**<.001** *	230.79 (44.26‐1203.52)	**<.001** *	38.46 (11.93‐123.99)	**<.001** *
Intermediate risk (score = 2‐3) n = 33	4.24 (1.70‐10.59)	**.002** *	2.42 (1.24‐4.72)	**.010** *	3.64 (1.43‐9.28)	**.007** *	2.15 (1.09‐4.27)	**.028** *
Good risk (score = 0‐1) n = 25	1		1		1		1	

Bold values are statistically significant with *α* < 0.05. Abbreviations: CI, confidence interval; HR, hazard ratio; OS, overall survival; PFS, progression free survival; UVA, univariable analysis.

a MVA controlled for age, race, sex, number of prior lines of therapy, number of sites of metastasis and smoking status.

* Statistical significance at α < 0.05 by Chi‐square test**.**

**Figure 1:**
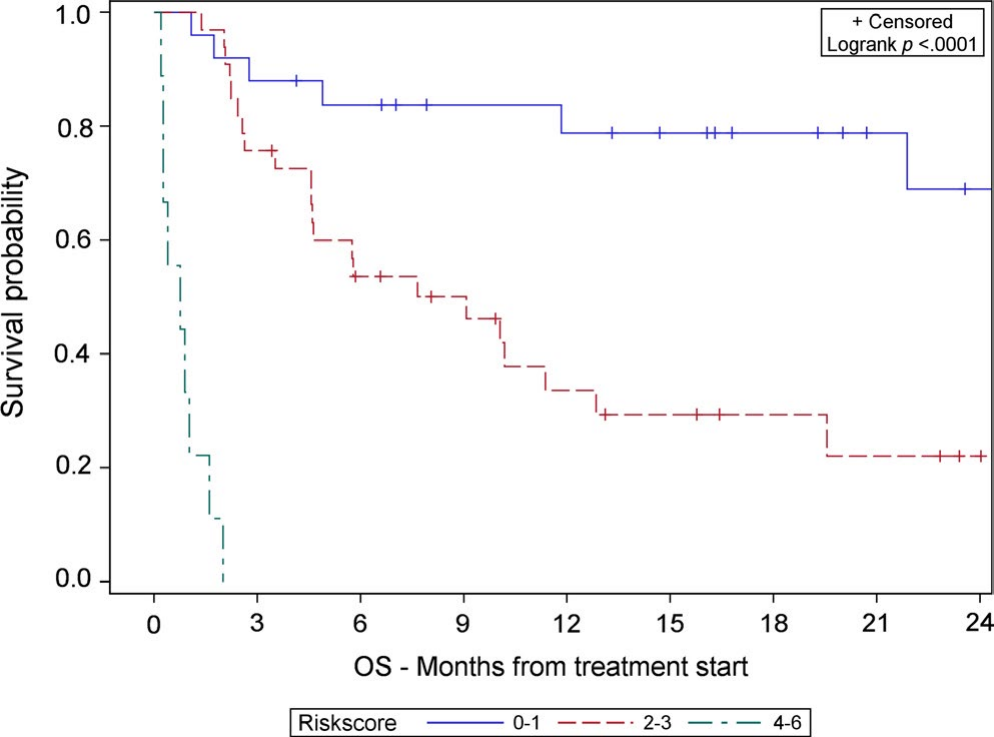
Kaplan‐Meier association of risk score and overall survival

**Figure 2:**
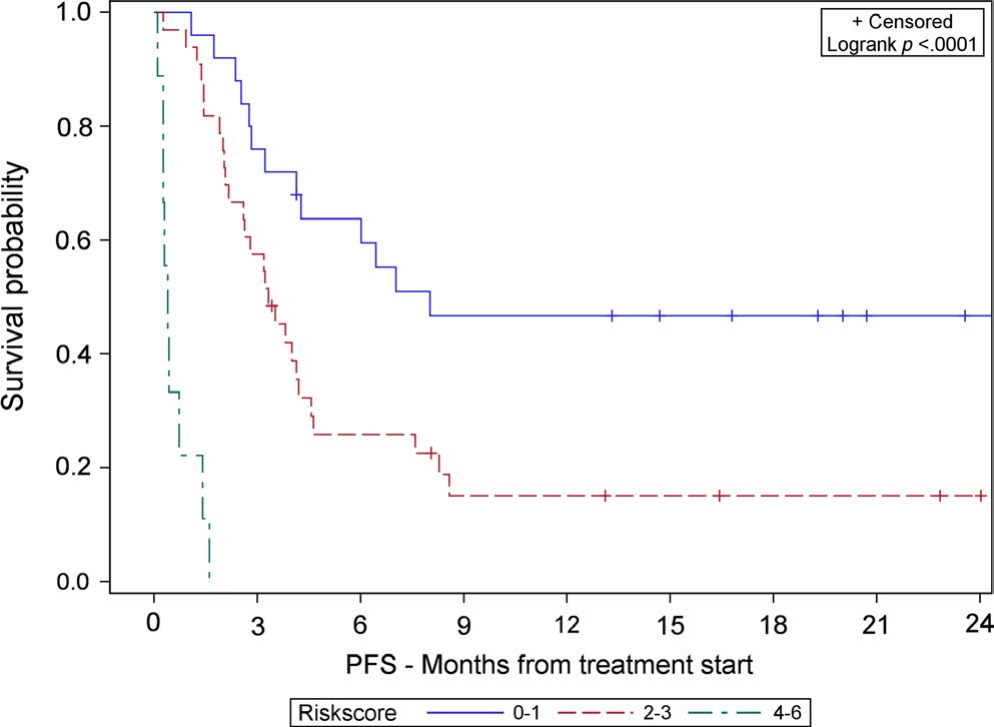
Kaplan‐Meier association of risk score and progression free survival

The median OS (Figure 1) and PFS (Figure 2) were significantly shorter for poor risk patients than intermediate risk and good risk patients per Kaplan‐Meier estimation. The median OS and PFS were 0.8 months and 0.4 months for poor risk patients, respectively, compared to the median OS of 9.1 months and median PFS of 3.3 months for intermediate risk patients. Median OS was not reached for good risk patients and median PFS was 8 months (all *P* < .0001). The Uno's C‐statistics for the final 3‐level risk group was 0.74 (Standard Error = 0.047) for OS and 0.70 (Standard Error = 0.043) for PFS.

High NLR, MLR, and PLR were associated with worse clinical outcomes in UVA and furthermore were correlated with one another (all Pearson correlation coefficients ≥0.45, all *P* ≤ .0001) (Figure S1). Given the correlation between these markers of inflammation, supplemental risk groups were created using NLR or MLR as a risk factor. Supplemental Kaplan‐Meier estimates for NLR‐based risk groups and association with OS and PFS are given in Figure [Link], [Link]A,B, respectively. The optimal cut for NLR was 4.67. Supplemental Kaplan‐Meier estimates for MLR‐based risk groups and association with OS and PFS are given in Figure [Link], [Link]A,B, respectively. The optimal cut for MLR was 0.545.

## 4 DISCUSSION

The ability to predict survival of UC patients is helpful in guiding management and treatment options. As the number of FDA‐approved ICI treatments for UC patients grows, the urological oncology field would benefit from an all‐encompassing risk scoring system for UC patients receiving ICI. In this study, we developed a hypothesis‐generating risk scoring system based on four variables to represent systemic inflammation (PLR), tumor microenvironment (liver metastasis), nutritional status (albumin), and clinical presentation (ECOG PS). Each of these variables is readily available in the clinical setting, thus, increasing the practicality and usefulness of the risk scoring system.

Bellmunt et al (2010) developed a scoring system for UC patients who experience treatment failure with platinum‐based regimens.^15^ Their scoring system included three factors: ECOG PS, hemoglobin level, and liver metastasis. Sonpavde et al (2016) later proposed five factors for prognosis of UC patients receiving salvage therapy: ECOG PS, liver metastasis, hemoglobin, time from prior chemotherapy, and albumin. Our scoring system validates three of these factors, adds a factor for assessing systemic inflammation, and is novel in its application to patients receiving ICI rather than chemotherapy.

Our finding that high PLR predicts poor clinical outcomes is consistent with and builds upon previous studies investigating the significance of markers of inflammation in patients receiving ICI. Baseline NLR, MLR, and PLR and early change in these variables have proven to be significantly associated with clinical outcomes in patients receiving ICI across several primary malignancies.^16^, ^17^, ^18^, ^19^, ^20^, ^21^ In this study, markers of inflammation are highly correlated with one another. With stepwise variable selection in building our risk scoring system, PLR was found to affect clinical outcomes most significantly, but with such high correlation among biomarkers of inflammation, PLR could be substituted by NLR or MLR. One hypothesis as to why these markers are effective in prediction of survival is that these variables may reveal an inability to increase lymphocytes as part of host immune activation. The host immune system plays a key role in the success of ICI.^22^ High NLR, MLR, and PLR indirectly reflect immune dysregulation in these patients and suggest poor response to ICI treatment. The results of our analysis support the inclusion of an inflammatory biomarker in a prognostic model for UC patients treated with ICI.

Metastasis to the liver has long been established as a predictor for decreased survival in cancer patients receiving chemotherapy.^23^, ^24^ Several studies have also implicated liver metastasis in decreased response to ICI‐based therapies in cancers such as melanoma, lung cancer, colorectal cancer, and bladder cancer.^25^, ^26^, ^27^, ^28^, ^29^, ^30^ The continued significance of liver metastasis in cancer patients is provocative. This finding may be explained by the liver's role as a tolerogenic organ.^31^, ^32^, ^33^ Tumeh et al (2017) found that melanoma and lung cancer patients with liver metastases treated with anti‐PD‐1 agents had decreased CD8+T‐cell density at the liver metastatic margin and furthermore that these patients had decreased response rate and shortened PFS.^34^ The density of CD8+T‐cells in the liver may, therefore, affect response to ICI. Metastasis to the liver may disrupt the organ's immune‐modulatory role, thereby restricting the host's immune response to ICI‐based treatments. The physiology of the tumor microenvironment with liver metastasis should be further explored.

Our study found that both liver and bone metastases were poor prognostic factors in UC patients receiving ICI. Patients with bone or liver metastases had significantly shorter OS in UVA and patients with bone metastases also had significantly shorter PFS in UVA. Previous studies have shown that cancer patients of various primary malignancies with bone metastases have decreased survival.^35^, ^36^, ^37^, ^38^ The effect of bone metastases on survival of cancer patients receiving ICI is not well studied. One explanation for why bone metastases do not respond well to immunotherapy may be that transforming growth factor‐beta (TGF‐β) released from bone suppresses T‐cell proliferation and activity.^39^ Furthermore, TGF‐β production and the pool of regulatory T‐cells may increase under the influence of cancer cells.^40^, ^41^ The bone microenvironment in the setting of metastases, as a result, may inhibit the potential T‐cell antitumor responses that are essential for the efficacy of immunotherapy.^42^, ^43^ Although we did not include presence of bone metastasis as a risk factor in our model, updated prognostic models may consider including bone metastasis.

Our finding that low baseline albumin predicts shorter OS and PFS is consistent with previous data. Albumin transports hydrophobic species that are not otherwise soluble in plasma, including hormones, fatty acids, bilirubin, metals, and lipopolysaccharides.^44^, ^45^, ^46^ Albumin also binds to drugs, increasing their bioavailability.^47^ Levels of this protein reflect nutritional status and liver function. Low albumin has been correlated with incidence of morbidity and mortality in hospitalized patients 48 and baseline albumin has been explored as a predictor of survival in cancer patients, including in UC patients.^49^, ^50^, ^51^, ^52^, ^53^ Consequently, albumin levels may be an indicator of illness severity. Moreover, albumin may play an important regulatory role in the immune system.^54^, ^55^

The final prognostic factor included in the Emory Risk Scoring System for UC patients is ECOG PS, which is a widely used method for assessing functional status of cancer patients. A patient's ECOG PS can be used to predict their ability to tolerate therapy and has consistently shown to be highly correlated with survival, including in patients receiving ICI.^56^, ^57^, ^58^ Determining a UC patient's ECOG PS requires no more than an assessment of their ability to perform activities of daily living (ADLs) and should be determined prior to treatment initiation. It stands to reason that a patient who cannot perform ADLs will not tolerate treatment as well as patients who are fully active, and therefore, patients with higher ECOG PS scores have worse clinical outcomes.

While the results of this study are clinically relevant, there are some limitations that should be mentioned. First, this is a retrospective study, which is vulnerable to selection bias. We tried to lessen the effects of this potential bias by including all UC patients who received at least one dose of ICI at our site, regardless of the treatment regimen or other baseline characteristics. Second, it is possible that metastasis to another site that we did not analyze could be more predictive of clinical outcomes than liver metastases. To minimize this problem, we categorized the five most common sites of metastasis in initial data collection and analyzed each site independently. Finally, the size of our cohort limits the statistical power of our findings. Future studies are necessary to validate the results of this study and to elucidate the underlying physiology explaining how systemic inflammation, nutritional status, clinical presentation and metastatic sites impact host immune response to ICI. Further efforts to develop prognostic models would likely be strengthened by the incorporation of tumor genomic data as well.

## 5 CONCLUSION

In this study, we developed a novel risk scoring system to predict clinical outcomes in UC patients receiving ICI therapy. We showed that increased baseline PLR, low baseline albumin, metastasis to the liver, and higher ECOG PS were associated with decreased survival in UC patients treated with ICI.

The results of this study reveal an important area for improvement in ICI‐based therapies for UC patients who fall into the poor risk group. Advancements in treating these patients are needed. Categorization into the poor risk group should not necessarily preclude patients from receiving ICI‐based therapies. Rather, this stratification may be useful in guiding treatment for these UC patients and may suggest that these patients should receive novel combination therapy, for example, more than one ICI or ICI with monoclonal antibodies targeting costimulatory molecules.^59^, ^60^

This is an important study in determining risk factors that affect clinical response of UC patients receiving ICI. This study is hypothesis‐generating and these findings warrant external validation with a larger, prospective study before being incorporated into clinical practice.

## CONFLICT OF INTEREST

BCC has a consulting/advisory role with Astellas Medivation, Pfizer, and Blue Earth Diagnostics and receives travel accommodations from Bristol‐Myers Squibb. MAB has a consulting/advisory role with Exelixis, Nektar, and Sanofi and receives research funding from Bayer, Bristol‐Myers Squibb, Genentech/Roche, Incyte, Nektar, AstraZeneca, Tricon Pharmaceuticals, Peleton, and Pfizer. MAB has acted as a paid consultant for and/or as a member of the advisory boards of Exelixis, Bayer, BMS, Eisai, Pfizer, AstraZeneca, Janssen, Genomic Health, Nektar, and Sanofi and has received grants to his institution from Xencor, Bayer, Bristol‐Myers Squibb, Genentech/Roche, Seattle Genetics, Incyte, Nektar, AstraZeneca, Tricon Pharmaceuticals, Peleton Therapeutics, and Pfizer for work performed as outside of the current study. All other authors declare no conflict of interest.

## AUTHOR CONTRIBUTIONS

JMS was involved in conceptualization, data curation, formal analysis, methodology, project administration, writing‐original draft, and writing‐review and editing. DJM was involved in data curation, formal analysis, writing‐original draft, and administration. YL was involved data curation, formal analysis, project administration, validation, visualization, and writing‐review and editing. MAB was involved in conceptualization, formal analysis, funding acquisition, investigation, project administration, and writing‐ review and editing. DR, JB, EEH, GAR, SC, HK, MA, KO, WBH, VAM, OK, and BCC were involved in investigation, resources, and writing‐ review and editing. All authors reviewed and approved the final version of the manuscript and agree to be accountable for all aspects of the work in ensuring that questions related to the accuracy or integrity of any part of the work are appropriately investigated and resolved.

## Data Availability

The datasets used and/or analyzed during the current study are available from the corresponding author on reasonable request.
