# Supplementary figures and images for: Novel risk group stratification for metastatic urothelial cancer patients treated with immune checkpoint inhibitors
Source: Cancer Med. 2020 Feb 25;9(8):2752–60. doi: 10.1002/cam4.2932 (PMC7163104; doi:10.1002/cam4.2932)

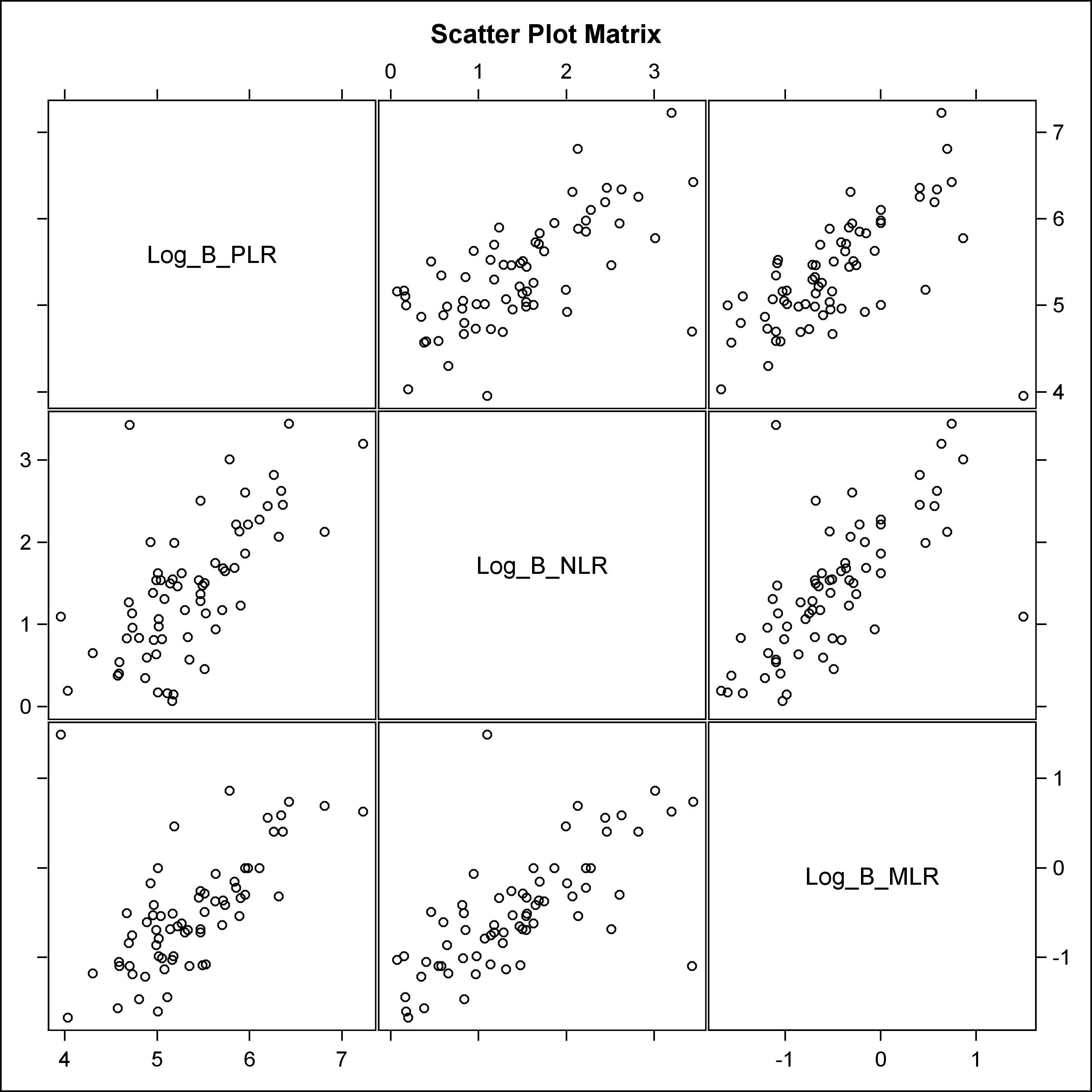

Supplement: Supplementary file 1 [file CAM4-9-2752-s001.png]

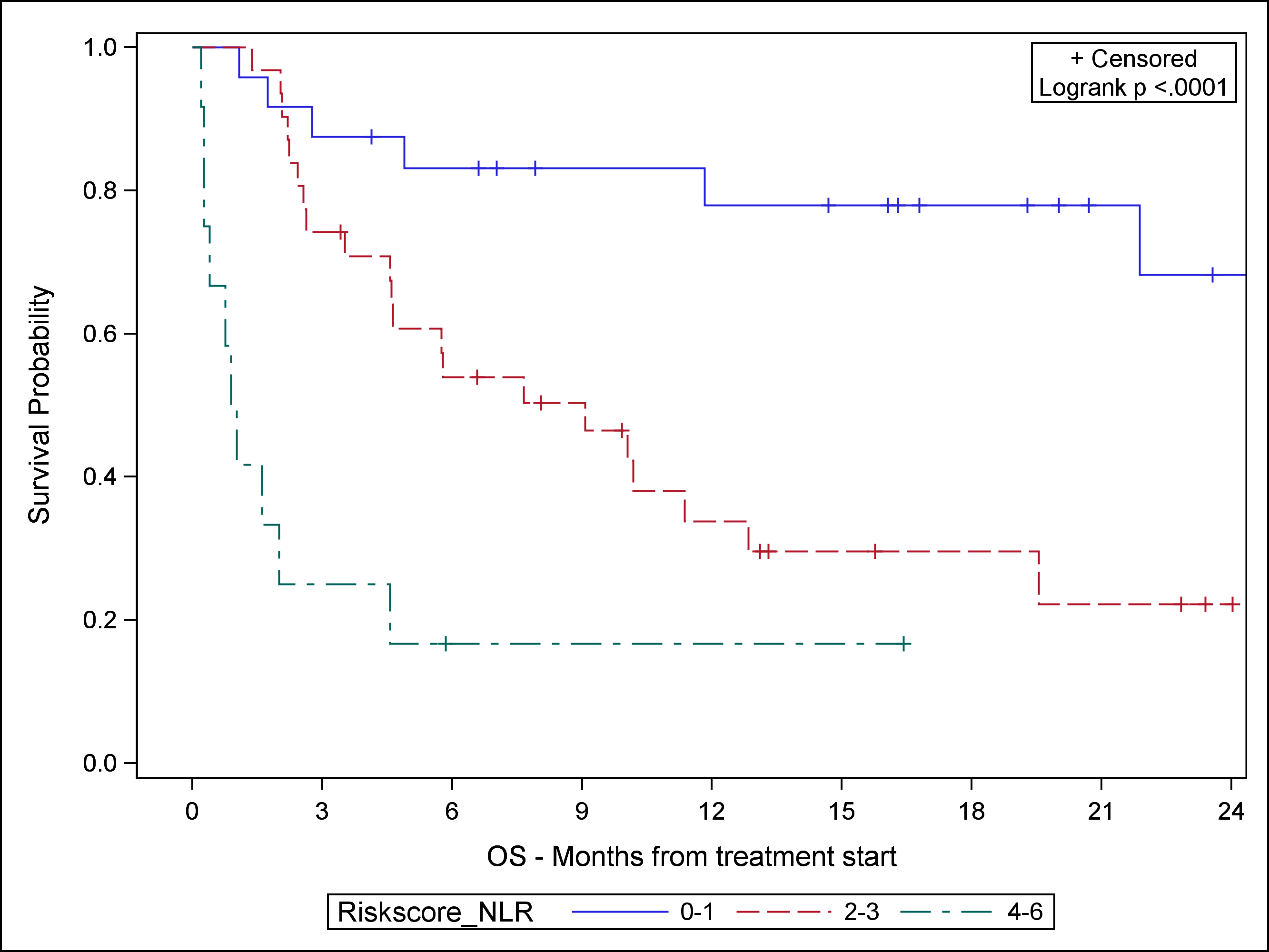

Supplement: Supplementary file 2 [file CAM4-9-2752-s002.png]

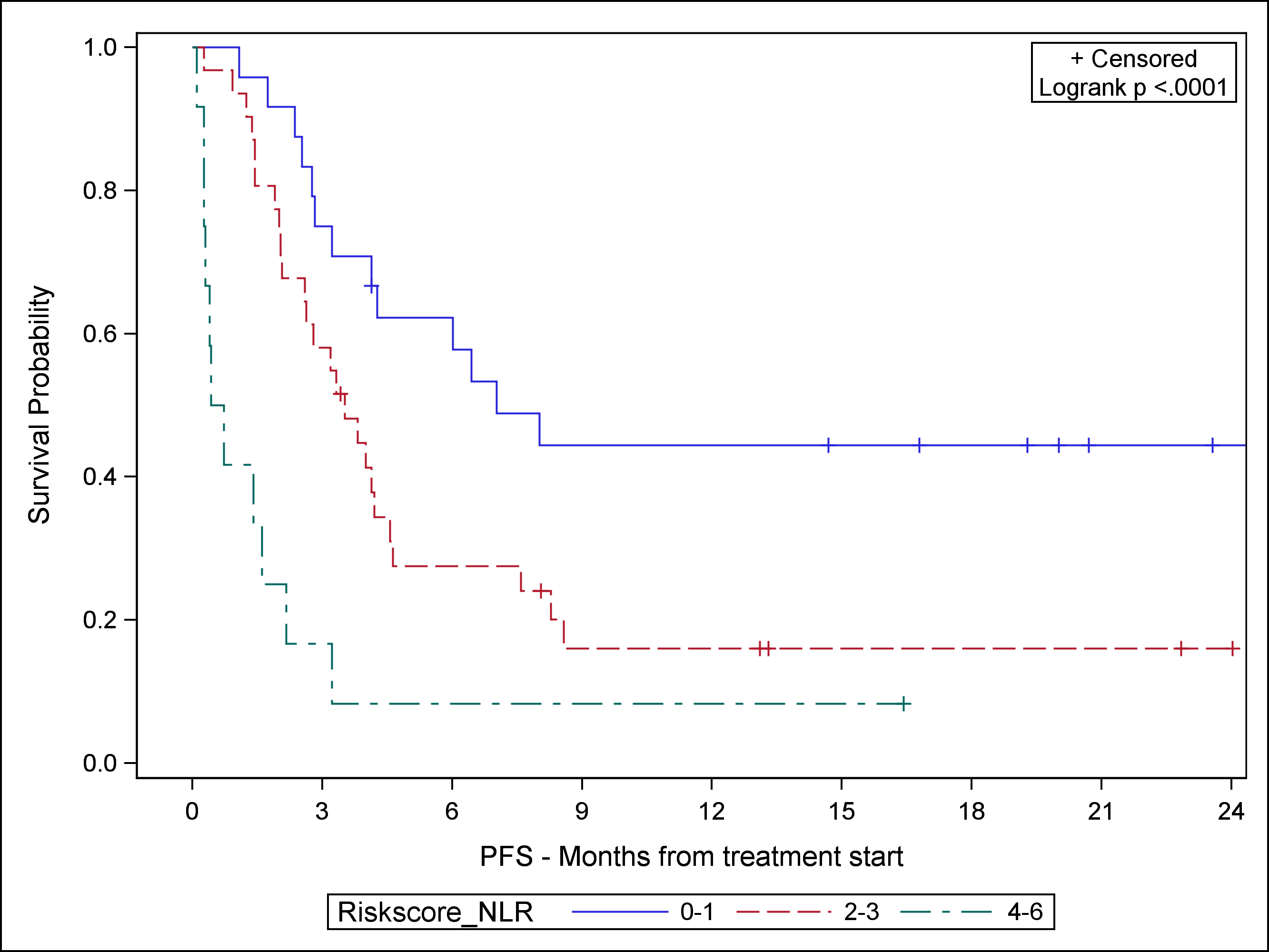

Supplement: Supplementary file 3 [file CAM4-9-2752-s003.png]

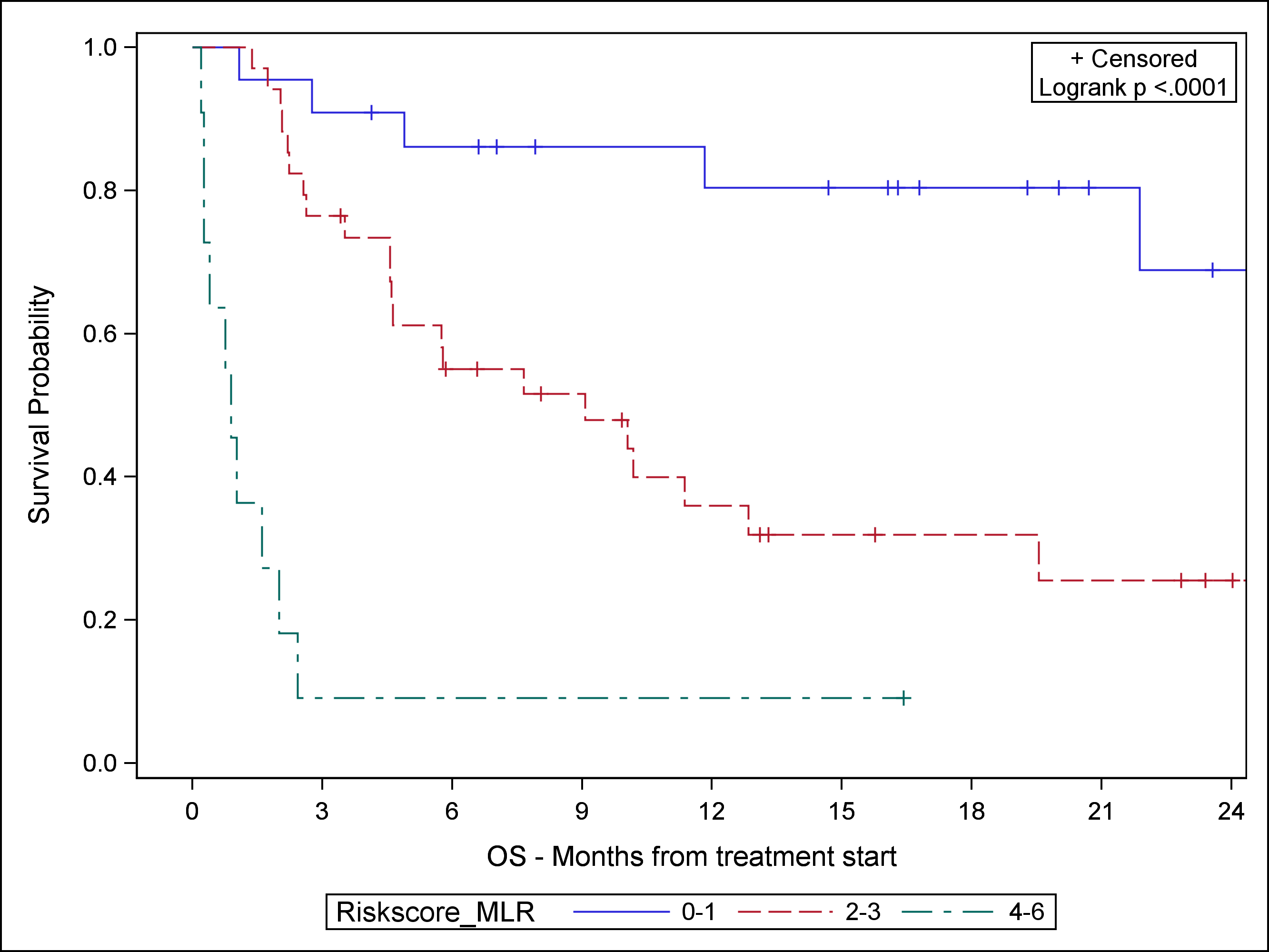

Supplement: Supplementary file 4 [file CAM4-9-2752-s004.png]

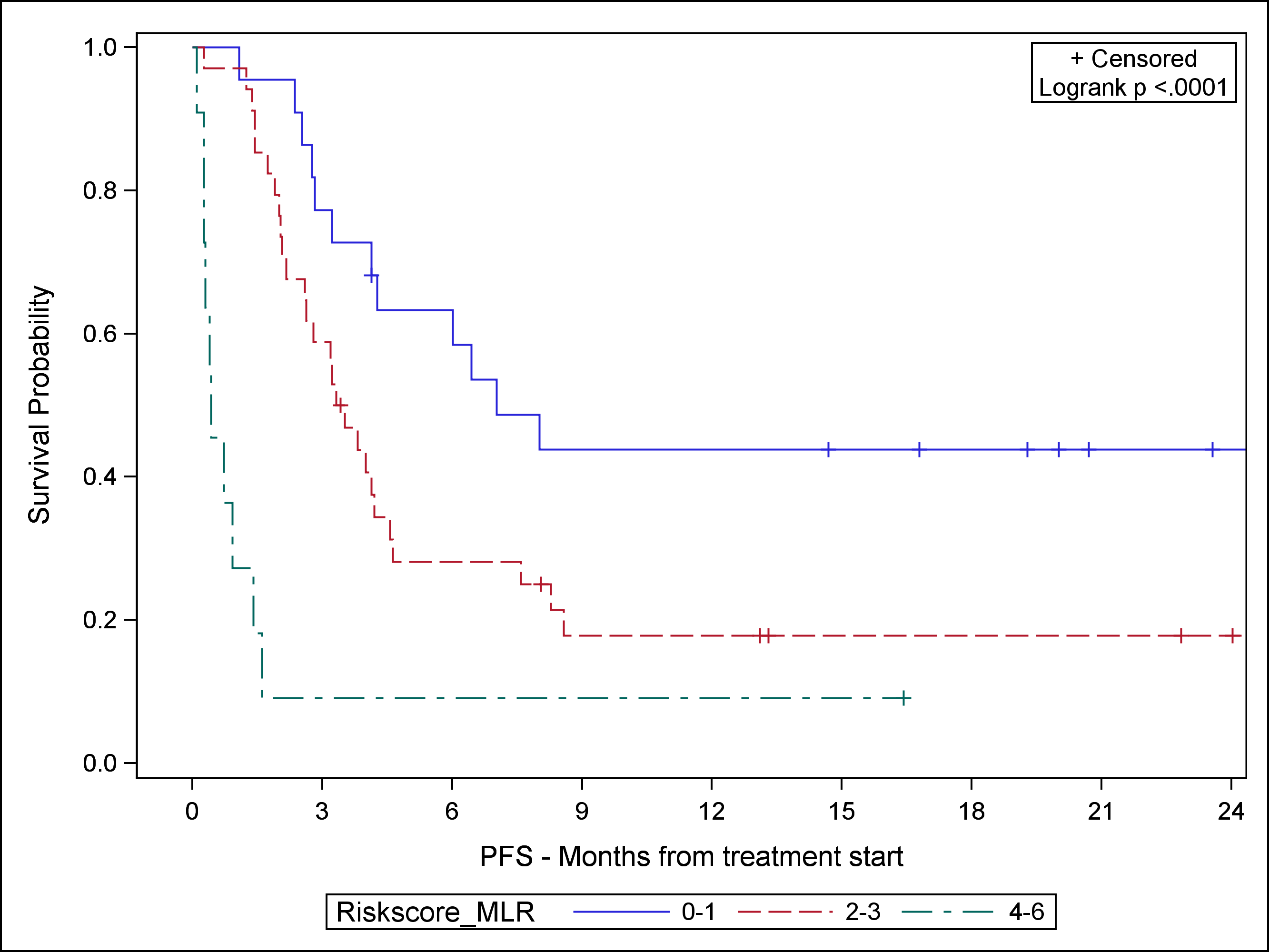

Supplement: Supplementary file 5 [file CAM4-9-2752-s005.png]
