# Supplementary material for: Novel risk group stratification for metastatic urothelial cancer patients treated with immune checkpoint inhibitors
Source: Cancer Med. 2020 Feb 25;9(8):2752–60. doi: 10.1002/cam4.2932 (PMC7163104; doi:10.1002/cam4.2932)
Supplement: Supplementary file 6 [file CAM4-9-2752-s006.docx]

**Supplementary Information**

**Supplemental Figure 1: Pearson Correlation of NLR, MLR, and PLR**

**Supplemental Figure 2A: Kaplan-Meier Association of NLR-Based Risk Score and OS**

**Supplemental Figure 2B: Kaplan-Meier Association of NLR-Based Risk Score and PFS**

**Supplemental Figure 3A: Kaplan-Meier Association of MLR-Based Risk Score and OS**

**Supplemental Figure 3B: Kaplan-Meier Association of MLR-Based Risk Score and PFS**
